# A study of the desulfurization selectivity of a reductive and extractive desulfurization process with sodium borohydride in polyethylene glycol

**DOI:** 10.1038/s41598-020-67235-8

**Published:** 2020-06-26

**Authors:** Xianglong Meng, Pin Zhou, Lu Li, Lizhong Liu, Mingming Guo, Tonghua Sun

**Affiliations:** School of Environmental Science and Engineering, Shanghai Jiao Tong University, Dongchuan Road 800, Shanghai, 200240 China

**Keywords:** Environmental chemistry, Pollution remediation, Natural hazards

## Abstract

The selectivity of a facile reductive and extractive desulfurization process was studied. In this desulfurization method, polyethylene glycol was used as the extractant, and sodium borohydride was used as the reductant. Several different simulated fuels were prepared by dissolving thiophenic sulfides, methylbenzene and hexylene in octane. The results showed that methylbenzene and olefins had different effects on different sulfur compounds during this desulfurization process. The extraction and reduction mechanisms were also explained. Four factors could affect the desulfurization performance: (1) intermolecular hydrogen bonding: (a) active O bonding with aromatic H or (b) S bonding with H atoms in hydroxide radicals, (2) “like-dissolves-like” interactions between polyethylene glycol and thiophenic sulfides, (3) the methyl steric hindrance effect and the electron density of sulfur atoms, and (4) the combination of S atoms with produced nickel boride to form active desulfurization centres. The desulfurization reaction path was also deduced according to the GC/MS results.

## Introduction

Refined fuels still contain many sulfur compounds, especially organosulfur compounds^1^. During fuel combustion, the sulfur compounds are oxidized to sulfur oxides and released into the air. Sulfur oxides can cause serious problems, such as acid rain, air pollution and the poisoning of metal catalysts in automobiles^2,3^. Therefore, many countries worldwide have set strict standards to control the sulfur content of fuels, and some countries even demand that the sulfur content be reduced to near-zero levels (<10 ppm)^4^. To comply with this requirement, many desulfurization technologies have been studied by researchers worldwide, such as hydrodesulfurization (HDS), oxide desulfurization (ODS)^5,6^, extractive desulfurization (EDS)^7,8^, absorptive desulfurization^9,10^, bio-desulfurization^11^ and electrochemical desulfurization^12,13^.

Among the methods illustrated above, HDS is the most mature desulfurization method in industrial production, but there are still some problems waiting to be solved. HDS is less effective for sulfides containing aromatic rings and their derivatives than for other compounds. In addition, HDS requires a high investment and harsh operating conditions^4^. Furthermore, another important problem is that during the HDS process, hydrogenation of benzene and olefins will take place, which will decrease the fuel octane number. As a result, it is imperative to improve the selectivity of the HDS process to remove the maximum amount of sulfur compounds and minimize olefin hydrogenation (OHYD)^14,15^. Li *et al*. studied a sulfided CoMo/SiO_2_ compound that exhibited high hydrodesulfurization selectivity^16^. Nikulshin *et al*. synthesized a series of CoMo/Al_2_O_3_ compounds and tested their reactivity during the HDS process. The results showed that the HDS selectivity correlated linearly with the edge-to-corner ratio of CoMoS phases^17^. In our previous work, we found a new reduction and extraction desulfurization (REDS) method based on NaBH_4_ and NiCl_2_ in PEG that obtained a high desulfurization efficiency for thiophenes at atmospheric temperature and pressure. In this research, we also studied the selectivity of this REDS method. FCC gasoline contains many compounds, such as alkenes (24%) and aromatic hydrocarbons (36%). Therefore, an additive reaction of C = C may also happen during this reductive desulfurization process. As a result, selectivity is an important evaluation index of this REDS method. Furthermore, thiophenic sulfides and aromatic compounds have similar chemical structures, which may affect the solubility of thiophenic sulfides in PEG, so it is necessary to explore the influence of the benzene series on this desulfurization method. In this research, different simulated fuels were prepared by dissolving hexylene, methylbenzene and organic sulfur compounds in octane. The desulfurization performance of this desulfurization method for different simulated fuels was detected. The extraction and reduction mechanisms were also determined.

## Results

### The effect of sulfur concentration on desulfurization efficiency

**Figure 1:**
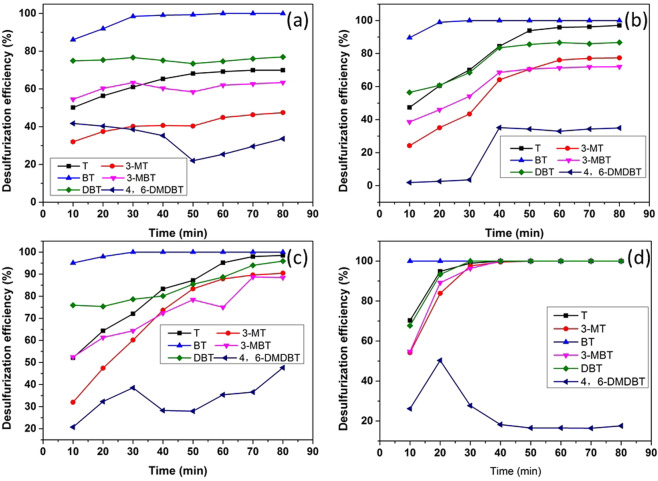
Effect of sulfur concentration on desulfurization efficiency: (**a**) T, 3-MT, BT, 3-MBT, DBT and 4,6-DMDBT, all 166.7 ppm, (**b**) sulfur content of 83.3 ppm, (**c**) sulfur content of 55.5 ppm, and (**d**) sulfur content of 41.67 ppm. Other reaction conditions: NaBH_4_/S molar ratio = 12, NiCl_2_/S molar ratio = 3, PEG/oil volume ratio = 1, reaction time = 80 min, stirring speed = 700 rpm, RT.

The initial sulfur concentration will affect the desulfurization capability of various technologies. When the sulfur content increases, some desulfurization processes may not produce high-quality gasoline. Most studies have investigated fuels with a sulfur content of up to 500 ppm. In this research, model fuels were prepared by dissolving T, 3-MT, BT, 3-MBT, DBT and 4,6-DMDBT in octane with sulfur contents of approximately 250, 333, 500, and 1000 ppm. The desulfurization results are shown in Fig. 1. The removal of BT and T was 100% or nearly 100% when the sulfur content was under 500 ppm, and the desulfurization efficiency of T decreased to 70% when the sulfur content increased to 1000 ppm. The desulfurization efficiency of 3-MT, 3-MBT and DBT decreased from 100%, 100%, and 100% to 47%, 63% and 77%, respectively, as the sulfur content increased from 250 ppm to 1000 ppm. The desulfurization efficiency of 4,6-DMDBT was nearly unchanged as the sulfur content increased. As shown in Fig. 1, when the total sulfur content was 500 ppm, most of the sulfides could be removed. Therefore, this desulfurization process can be used for fuels with a sulfur content of approximately 500 ppm, and the subsequent experiment used only simulated fuel with a 500 ppm sulfur content.

### The selectivity of this process for different sulfur compounds

**Figure 2:**
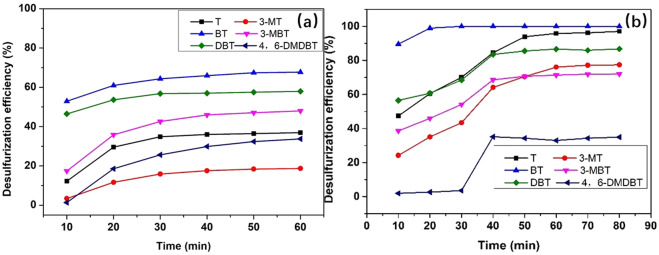
Desulfurization selectivity for different sulfides. Extraction desulfurization performance by PEG. PEG/oil volume ratio = 1, reaction time = 60 min SOI-1.

**Figure 3:**
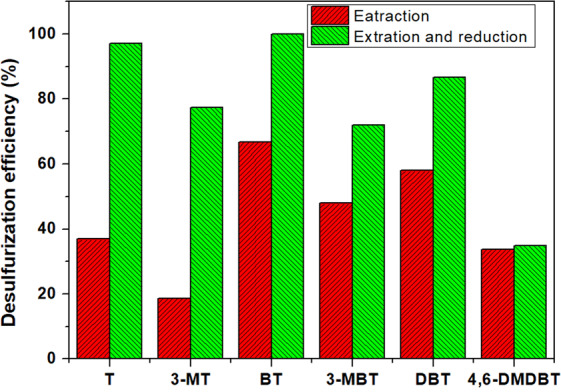
Comparison of desulfurization performance between the extractive desulfurization method and the REDS method.

**Figure 4:**
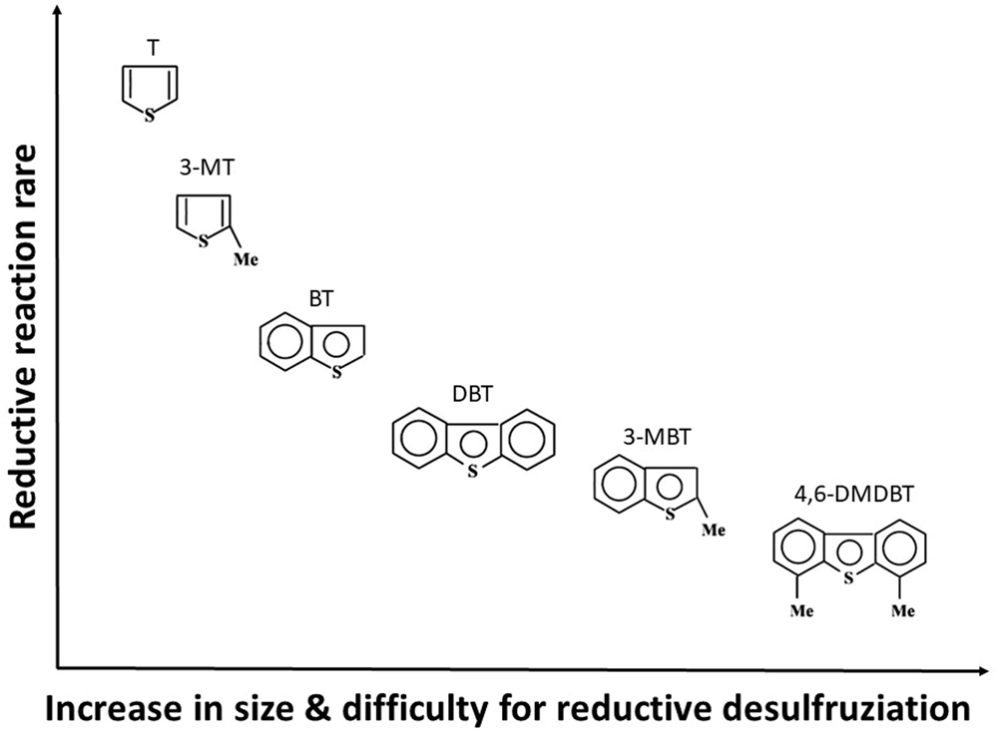
Difficulty of reductive desulfurization of different sulfides.

Different sulfur compounds have different electron densities on the sulfur atoms, and steric hindrance plays important roles in desulfurization reactions^18^. Therefore, it is necessary to research the desulfurization effect of this extractive and reductive desulfurization process on different sulfur compounds. In this research, a model fuel (SOI-1) composed of 83.5 ppm each of T, 3-MT, BT, 3-MBT, DBT, and 4,6-DMDBT was desulfurized at 288 K. The desulfurization efficiency of sulfur compounds vs. reaction time is shown in Fig. 1(b). The results showed that 100% BT was removed at 20 min in this system, and more than 97% T was removed after 80 min reaction. The desulfurization efficiencies of DBT, 3-MT, 3-MBT and 4,6-DMDBT were 87%, 77%, 72% and 35%, respectively. The desulfurization efficiency was found to decrease in the order of BT, T, DBT, 3-MT, 3-MBT and 4,6-DMDBT. The desulfurization performance of extraction by PEG was also investigated, and the results are shown in Figs. 2 and 3. The K_N_ values of different sulfur compounds are shown in Table 1. The K_N_ values of different sulfur compounds obtained by PEG extraction follow the order BT > DBT > 3-MBT > T > 4,6-DMDBT > 3-MT. The reduction desulfurization amount was obtained by subtracting the extraction desulfurization efficiency from the REDS desulfurization efficiency. It could be deduced that the difficulty of the reductive reaction was in the order 4,6-DMDBT > 3-MBT > DBT > BT > 3-MT > T, as shown in Fig. 4. The reduction reactivity differences between different sulfides could be ascribed to the steric hindrance caused by methyl groups and the electron density on sulfur atoms^19^. These effects made the interaction of sulfur atoms with the reductant or catalyst active sites more difficult.

**Table 1:** K_N_ values of different sulfides obtained by PEG extraction.

Sulfur compound	T	3-MT	BT	3-MBT	DBT	4,6-DMDBT
K_N_	0.59	0.23	2.10	0.92	1.40	0.51

### Effect of olefin on desulfurization efficiency

**Figure 5:**
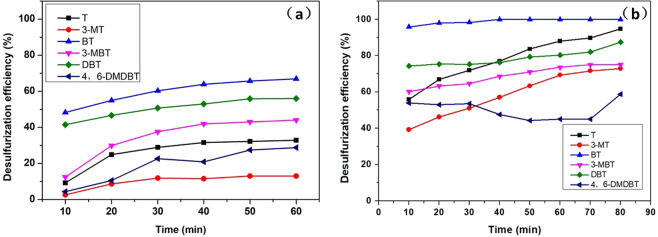
Effect of hexylene on desulfurization efficiency. (**a**) Extraction desulfurization performance, SOI-2, reaction time = 60 min. (**b**) REDS performance. Other reaction conditions: NaBH_4_/S molar ratio = 12, NiCl_2_/S molar ratio = 3, PEG/oil volume ratio = 1, reaction time = 80 min, stirring speed = 700 rpm, RT, SOI-2.

A simulated fuel was prepared by dissolving hexylene (approximately 24%) in octane, and the desulfurization efficiency is shown in Fig. 5 and Table 2. The desulfurization performance in Fig. 5(b) changed compared with that in Fig. 1(b). The removal of BT was unaffected by hexylene, and a 100% desulfurization efficiency was maintained. This is because BT was much easier than the other sulfur compounds to extract from the simulated fuel. Hexylene had little effect on the desulfurization efficiency of T, 3-MT and DBT, and the desulfurization efficiency decreased by approximately 2.3%, 4.46% and 0.24%, respectively. Furthermore, products of hexylene were not detected by GC/MS, as shown in the discussion section, and hexylene was not reduced by NaBH_4_. Therefore, hexylene did not waste H* and would not have affected the sulfur reduction reaction. The reason for the decrease in desulfurization efficiency may be that hexylene changed the extraction performance of PEG. As Fig. 5(a) and Table 2 show, the desulfurization efficiency decreased obviously compared with the results in Fig. 2. Therefore, hexylene had an effect on the extraction performance of PEG.

**Table 2:** Decrease in desulfurization efficiency in Fig. 5 compared with the results in Figs. 1(b) and 2.

Sulfur compound	T	3-MT	BT	3-MBT	DBT	4,6-DMDBT
Decrease in desulfurization efficiency from Fig. 5 (b) to Fig. 1(b) (%)	2.3	4.46	0	−3.05	0.24	−13.18
Decrease in desulfurization efficiency from Fig. 5(a) to Fig. 2 (%)	4.08	5.73	0.80	3.90	1.95	4.92

### Effect of aromatics on desulfurization efficiency

**Figure 6:**
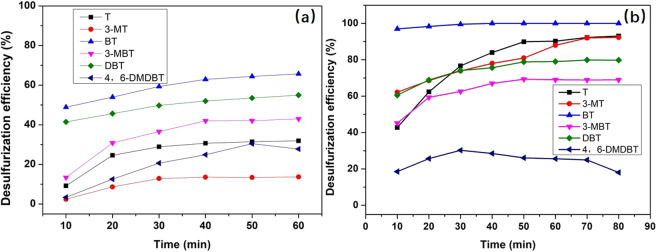
Effect of methylbenzene on desulfurization efficiency. (**a**) Extraction desulfurization performance, SOI-3, reaction time = 60 min. (**b**) REDS performance. Other reaction conditions: NaBH_4_/S molar ratio = 12, NiCl_2_/S molar ratio = 3, PEG/oil volume ratio = 1, reaction time = 80 min, stirring speed = 700 rpm, RT, SOI-3.

Another simulated fuel was also prepared by dissolving methylbenzene (approximately 36%) in octane to study the effect of benzene on the desulfurization efficiency of this method, and the results are shown in Fig. 6 and Table 3. As Fig. 6(a) and Table 3 show, methylbenzene led to decreases in extraction desulfurization efficiency of approximately 4.87%, 5.02%, 2.33%, 5.13%, 3.92% and 8.14% for the six sulfur compounds. It could be concluded that methylbenzene affected the PEG extraction performance. This is because aromatic H atoms in methylbenzene also interact with O atoms in PEG^20^. Figure 6(b) and Table 3 show the change in desulfurization efficiency of the REDS method. The desulfurization efficiency of BT was not affected by methylbenzene. The removal rates of T, 3-MBT, DBT and 4,6-DMDBT all decreased when the simulated oil was mixed with methylbenzene. The removal rate of 3-MT increased from 77% to 92%. The REDS method was also affected by methylbenzene, but less so than just the extraction method. Most importantly, the REDS method maintained a high desulfurization efficiency.

**Table 3:** Decrease in desulfurization efficiency in Fig. 6 compared with the results in Fig. 2 and Fig. 1(b).

Sulfur compound	T	3-MT	BT	3-MBT	DBT	4,6-DMDBT
Decrease in desulfurization efficiency from Fig. 6 (b) to Fig. 1(b) (%)	3.96	−14.91	0	3.01	6.89	9.93
Decrease in desulfurization efficiency from Fig. 6(a) to Fig. 2 (%)	4.87	5.02	2.33	5.13	3.92	8.14

### Effect of olefins and aromatics on desulfurization efficiency

**Figure 7:**
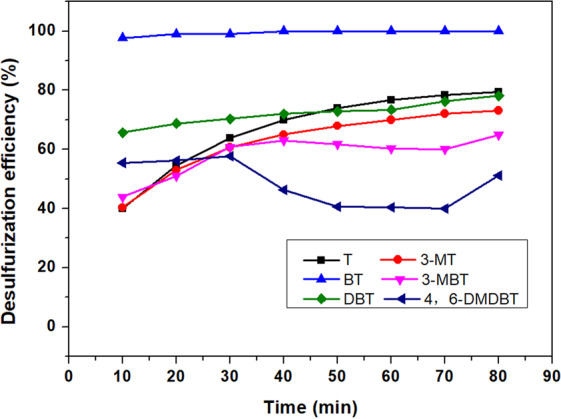
Effect of hexylene and methylbenzene on desulfurization efficiency. Other reaction conditions: NaBH_4_/S molar ratio = 12, NiCl_2_/S molar ratio = 3, PEG/oil volume ratio = 1, reaction time = 80 min, stirring speed = 700 rpm, RT, SOI-4.

A simulated fuel was prepared by dissolving methylbenzene (approximately 36%) and hexylene (approximately 24%) in octane, which was similar to the composition of the real FCC fuel. The desulfurization results for this simulated fuel are shown in Fig. 7 and Table 4. The desulfurization efficiency of BT was not affected by methylbenzene and hexylene. The removal rates of T, 3-MT, DBT and 3-MBT all decreased when the simulated oil was mixed with methylbenzene and hexylene. 4,6-DMDBT was difficult to remove, and a low desulfurization rate was difficult to maintain. When methylbenzene and hexylene were added together, the desulfurization efficiency decreased more than it did when just adding one component. This is because methylbenzene and hexylene dramatically reduced the amount of n-octane in the simulated oil. This reduction could change the polarity of the simulated oil and affect the extraction desulfurization efficiency. This effect does not change linearly with the proportion of oil components. Therefore, the desulfurization efficiency of SOI-4 was much lower than those of SOI-2 and SOI-3.

**Table 4:** Decrease in desulfurization efficiency in Fig. 7 compared with the results in Fig. 1(b).

Sulfur compound	T	3-MT	BT	3-MBT	DBT	4,6-DMDBT
Decrease in desulfurization efficiency (%)	17.55	4.33	0	7.08	8.63	−6.15

## Discussion

**Figure 8:**
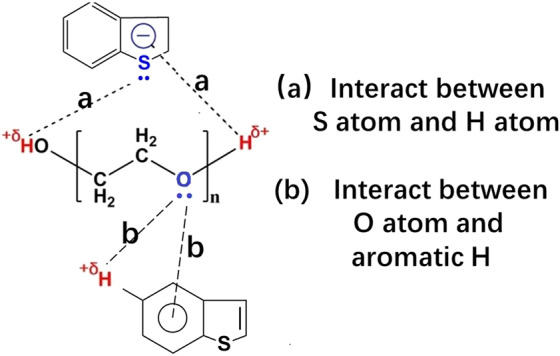
Extraction desulfurization mechanism.

PEG is rich in –OH groups and C-O-C groups, which provide a large number of active H atoms and active O atoms. As shown in Fig. 8, aromatic H in thiophenic sulfides can bond with active O on PEG^21^, so PEG can easily extract thiophenic sulfides from simulated fuel. The benzene ring in methylbenzene would also affect the extraction desulfurization performance, and the results of the desulfurization experiment in Fig. 6(b) show that methylbenzene has an effect on desulfurization efficiency but the effect was not significant. There are two reasons for this phenomenon. The most important reason was that S atoms could bond with the produced NiB in PEG; furthermore, this portion of S atoms could be reduced by active H produced by NaBH_4_. Therefore, thiophenic sulfides could be continuously reduced and continuously extracted by PEG. Another reason was that there was another interaction between the active H atom of PEG and the S atom of thiophenic sulfides, which made sulfur compounds easier to extract with PEG than methylbenzene.

**Figure 9:**
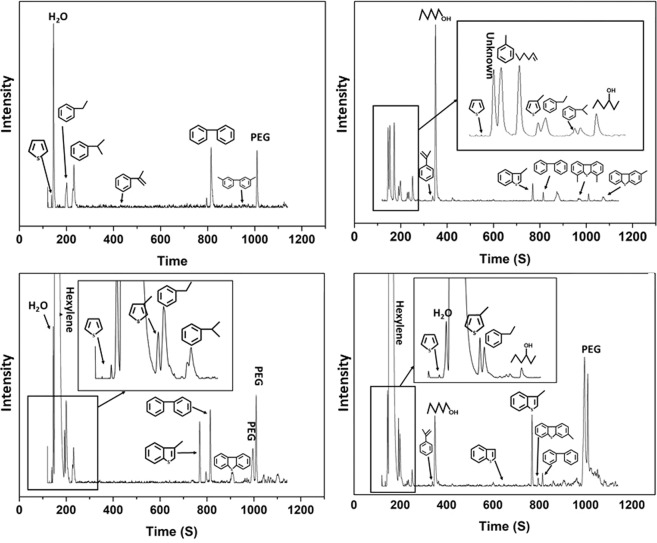
GC/MS spectra of model fuels after the REDS process: (**a**) SOI-1, (**b**) SOI-2, (**c**) SOI-3, (**d**) SOI-4.

**Figure 10:**
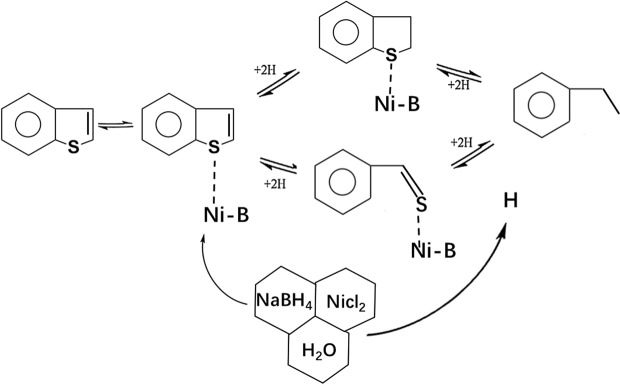
Reductive desulfurization reaction mechanism.

The components in the simulated fuels after desulfurization were detected by GC/MS. As shown in Fig. 9, after the REDS process, most of the organosulfur compounds were eliminated, mainly biphenyl (BP), propylbenzene (PB) and ethylbenzene (EB) were present in the simulated fuels after desulfurization, and no reduction products of methylbenzene or olefins were detected in the desulfurized fuel. Therefore, it could be concluded that the REDS process could not reduce methylbenzene, olefins or the benzene ring of thiophenic sulfides. Figure 10 shows the reduction desulfurization mechanism; the desulfurization reaction path is explained with BT as an example. NaBH_4_ could react with NiCl_2_ to form Ni-B and active hydrogen. Ni-B is a desulfurization activity centre, and it can combine with thiophenic sulfides and use the generated active hydrogen to complete the catalytic reduction desulfurization reaction.

## Methods

### Materials

NaBH_4_ (96%, AR) and nickel chloride hexahydrate (NiCl_2_.6H_2_O, > 98%, AR) were purchased from Sinopharm Chemical Reagent Co. Ltd (Shanghai, China). T, 3-MT, BT, 3-MBT, DBT and 4,6-DMDBT were purchased from Aladdin Reagent Co. Ltd (Shanghai, China). PEG-200, hexylene, octane and methylbenzene were purchased from Aladdin Reagent Co. Ltd (Shanghai, China).

### Desulfurization process and analytical methods^22^

Simulated fuels with a sulfur content of 500 ppm were prepared by dissolving thiophene (T), 3-methylthiophene (3-MT), benzothiophene (BT), 3-methylbenzothiophene (3-MBT), dibenzothiophene (DBT) and 4,6-dimethyldibenzothiophene (4,6-DMDBT) in different solvents made by mixing several components (e.g., hexylene, octane, and methylbenzene) in different percentages, as shown in Table 5.

**Table 5:** Components of different simulated oils (SOIs).

Components	Sulfur content (ppm)	Methylbenzene (%)	Hexylene (%)
SOI-1	500	—	—
SOI-2	500	—	24
SOI-3	500	36	—
SOI-4	500	36	24

A typical desulfurization experiment was carried out in a sealed two-neck flask under ambient conditions. NaBH_4_ (NaBH_4_/S molar ratio = 12), model fuel and PEG were added into the two-neck flask in turn with stirring. Then, NiCl_2_·6H_2_O (NiCl_2_/S molar ratio = 3) was slowly dripped into the mixture. After the reaction, the oil phase and PEG were separated by a separatory funnel. The sediment was separated from furfuryl alcohol by filtration and digested with hydrochloric acid. The desulfurization efficiency was calculated by the following formula (1):

Desulfurization efficiency (wt. %) = (TS_1_−TS_2_)/TS_1_*100% (1)

where TS_1_ is the total sulfur (TS) content in the original simulated fuel and TS_2_ is the TS content in the simulated fuel after desulfurization.

### Analytical methods

The sulfur (S) content in model gasoline after desulfurization was determined by a gas chromatograph-flame photometric detector (GC 7890B FPD, Agilent, USA) equipped with an HP-5 column (0.5 mm × 30 m). The analysis conditions were as follows: the injector temperature was 340 °C, the detector temperature was 250 °C, and the column temperature was programmed from 50 °C to 180 °C at 20 °C/min. The injection amount was 1 µL for all samples, and the split ratio was 90:1. The components of the model fuel after desulfurization were analysed by a gas chromatograph/mass spectrometer (GC/MS, 7890A-5975C, Agilent, USA) equipped with a DB-5MS column (30 m × 0.25 mm × 0.25 µm) with the following conditions: helium was used as the carrier gas at a constant flow of 1 mL/min. The injector temperature was 300 °C, and the oven temperature was programmed from 20 °C to 200 °C at 20 °C/min. The injection amount was 1 µL for all samples, and the split ratio was 10:1. The mass spectra conditions included an ionization voltage of 70 eV, ion source temperature of 230 °C, quadrupole temperature of 150 °C, and full scan mode in the m/z range of 20–400^22,23^.

## Conclusion

In conclusion, the REDS process is an efficient fuel desulfurization method. Although methylbenzene and hexylene had some effect on the desulfurization performance, the REDS process could selectively reduce thiophenic sulfur. Aromatic compounds and olefins mainly affect the sulfide extraction process by PEG. The selectivity of the REDS process for sulfides, aromatics and olefins was affected by four factors: (1) intermolecular hydrogen bonding: (a) active O bonding with aromatic H and (b) S atom bonding with H in hydroxide radicals, (2) “like-dissolves-like” interactions between polyethylene glycol and thiophenic sulfides, (3) the methyl steric hindrance effect and the electron density on sulfur atoms, and (4) the combination of S atoms with produced nickel boride to form active desulfurization centres.
